# Google Trends can improve surveillance of Type 2 diabetes

**DOI:** 10.1038/s41598-017-05091-9

**Published:** 2017-07-10

**Authors:** Nataliya Tkachenko, Sarunkorn Chotvijit, Neha Gupta, Emma Bradley, Charlotte Gilks, Weisi Guo, Henry Crosby, Eliot Shore, Malkiat Thiarai, Rob Procter, Stephen Jarvis

**Affiliations:** 1 Warwick Institute for the Science of Cities, University of Warwick, Coventry, CV4 7AL UK; 2 Department of Computer Science, University of Warwick, Coventry, CV4 7AL UK; 3 School of Engineering, University of Warwick, Coventry, CV4 7AL UK; 4 The Alan Turing Institute, The British Library, London, NW1 2DB UK; 5 Experian, The Sir John Peace Building, Experian Way, NG2 Business Park, Nottingham, NG80 1ZZ UK

## Abstract

Recent studies demonstrate that people are increasingly looking online to assess their health, with reasons varying from personal preferences and beliefs to inability to book a timely appointment with their local medical practice. Records of these activities represent a new source of data about the health of populations, but which is currently unaccounted for by disease surveillance models. This could potentially be useful as evidence of individuals’ perception of bodily changes and self-diagnosis of early symptoms of an emerging disease. We make use of the Experian geodemographic Mosaic dataset in order to extract Type 2 diabetes candidate risk variables and compare their temporal relationships with the search keywords, used to describe early symptoms of the disease on Google. Our results demonstrate that Google Trends can detect early signs of diabetes by monitoring combinations of keywords, associated with searches for *hypertension treatment* and *poor living conditions*; Combined search semantics, related to *obesity*, *how to quit smoking* and *improve living conditions (deprivation)* can be also employed, however, may lead to less accurate results.

## Introduction

Recent research demonstrates^1^ that more people in Britain are turning to Google to diagnose their ailments rather than going to see their doctor. Thus, according to Google UK^1, 2^, since 2008 there has been a surge in the number of people searching for information related to health, with 21.8 per cent of people in 2015 choosing to self-diagnose via search engines, rather than consulting their family, friends or even a professional GP.

It is reported that one of the main reasons why people are self-diagnosing online is they find it harder to get access to medical professionals^2^. According to a recent UK Digital Health Report^3^, which conducted research on 61 million Google searches and a survey of 1013 adults, 11 per cent of respondents prefer to self-diagnose online because they cannot get help from their doctor, while another 10.8 per cent said Google was the best option because their doctor was not available quickly enough^4^.

No consensus exists at the moment among medical practitioners in regards to the value of self-diagnosis. Thus, the British Medical Association recognizes that about one in five people are self-diagnosing online today and they are generally supportive of *bona fide* web resources like NHS Direct^5^. The Australian Patients Association, on the other hand, expresses concerns that more people tend to self-diagnose without seeking professional advice in the first instance and they advise against that very strongly because online information is often not to be relied upon^6^.

Although it is largely impossible to uncover the exact motives behind self-diagnosing online, researchers increasingly recognize that cases of *cyberhondria* (self-diagnosis by search engine) are predominantly based on the signs of real illness, leading people to fear the worst and seek remedies using easily accessible online channels^7^. Previous studies identified that such behaviours can be influenced by social, situational or psychological factors, including personal theories and beliefs^8, 9^ and vary with the availability of instruments for self-diagnosing, which may comprise online calculators and personal monitoring devices^10, 11^.

Despite wide media coverage and an increased recognition by health professionals of this trend towards self-diagnosis, there is currently a lack of understanding of how such personal disease detection information can be used to advance research towards accounting for the significance of self-diagnosis pathways on proactive health behaviours. In this paper, we use Type 2 diabetes as an example to investigate this phenomenon and its relationship with formal disease diagnosis mechanisms.

Type 2 diabetes is a growing public health problem associated with significant rates of mortality, morbidity and long-term financial healthcare costs^12^. This non-communicable disease, accounting for over 95% of diabetes cases worldwide^13^, is a result of complex gene-environment interactions; the role of several risks factors, including age, sex, ethnicity, family history, obesity, and hypertension is well documented in the research literature^14–17^. The precise interaction of these and other risk factors is complex, and varies both within and across populations^18^.

In order to identify diabetes risk with the highest degree of certainty, a number of complex diagnostic tests are required to be completed under clinical conditions. Such tests include physical examination, a urine sugar test, a urine ketones test, an oral glucose tolerance test, blood glucose tests (fasting plasma glucose and random plasma glucose), a C-peptide blood test or insulin level blood test^19^. Before a formal examination is scheduled, Diabetes UK recommends that the patient look for such symptoms as increased thirst, hunger or night-time urination, general fatigue, weight loss, blurred vision or sores that do not heal^20^.

As the early stages of Type 2 Diabetes do not usually exhibit signs or symptoms, the use of models for predicting risk of developing chronic disease have become more common in the last decade^21^. A recent systematic literature review revealed around 150 such models developed by epidemiologists and statisticians^13^, each designed to provide health information in an easily accessible format in order to enable planning, monitoring and coordination of clinical and public health interventions. Simple clinical models using readily available data can offer similar discrimination to more complex models using laboratory data or biomarkers, and clinical models that do not need clinical measurements may have a further utility in settings where clinical measurements are not available or are too costly to collect^12, 22^. The simplicity of the structure of these weighted models, which can comprise up to ten risk factors, including age, sex, ethnicity, smoking habits, hypertensive symptoms and obesity, has also enabled their widespread publication on the Internet, as online calculators and risk modeling tools. These include, amongst others, Diabetes Risk Calculator^23^, ARIC Diabetes Risk Calculator^24^, QDiabetes^25^, UKPDS Risk Engine^26^, etc.

In this study, we make use of currently adopted risk factors as background information against which the relationships between medically diagnosed diabetes and self-diagnosis are established: we hypothesize that false positive and negative components of self-diagnosis can fine-tune the performance of broadly defined risk factors in contemporary models by introducing specific behavioural preferences of the populations considered at risk. The latter can be also regarded as more temporally dynamic risk factors, currently unaccounted for in Type 2 diabetes models and, which, as a consequence, have been the cause of long-standing skepticism towards contemporary surveillance programs for chronic conditions^27^. In this study we assume that self-diagnosis is a quantifiable category, derived predominantly from traces of online human activity, while bearing in mind that not all self-diagnosing behaviour will be captured through digital records.

## Results

Worldwide, around 150 models are currently in use for Type 2 diabetes surveillance, and whilst broad areas of risk have been outlined (e.g., Gender, Age, Ethnicity, Deprivation, etc.), no definite consensus currently exists on the final inventory of the candidate risk variables^21^. It has been already recognized^28, 29^ that using new or alternative data sources for monitoring or in supporting public health decision-making often necessitates an understanding of the complex connections between time varying public health problems and the time varying signal of new data streams. The research literature demonstrates that such connections can be highly situational and depend on user behaviour or the influence of external forces on user behaviour^30^. In the context of this data-driven experiment we therefore proposed an evaluation of two model scenarios, one *with* and one *without* a self-diagnosis variable, on the matter of co-occurrence of their associated regressors with the public interest in the topic of diabetes on the web. This idea is linked to the so-called *post-GFT* (*Google Flu Trends*) *syndrome*, where several authors have argued for the need to [quantitatively] account for the *reasons* behind changing user behaviour when exploiting new data sources in the design of risk surveillance systems for anomalous events^31–34^. To take this idea further, in our study we aim to test the hypothesis that self-diagnosis, as a risk variable reflecting the dynamic nature of public interest in the topic, is significant for revealing additional behavioural and lifestyle risk factors, which are currently not accounted for in design of the digital disease detection tools.

**Figure 1:**
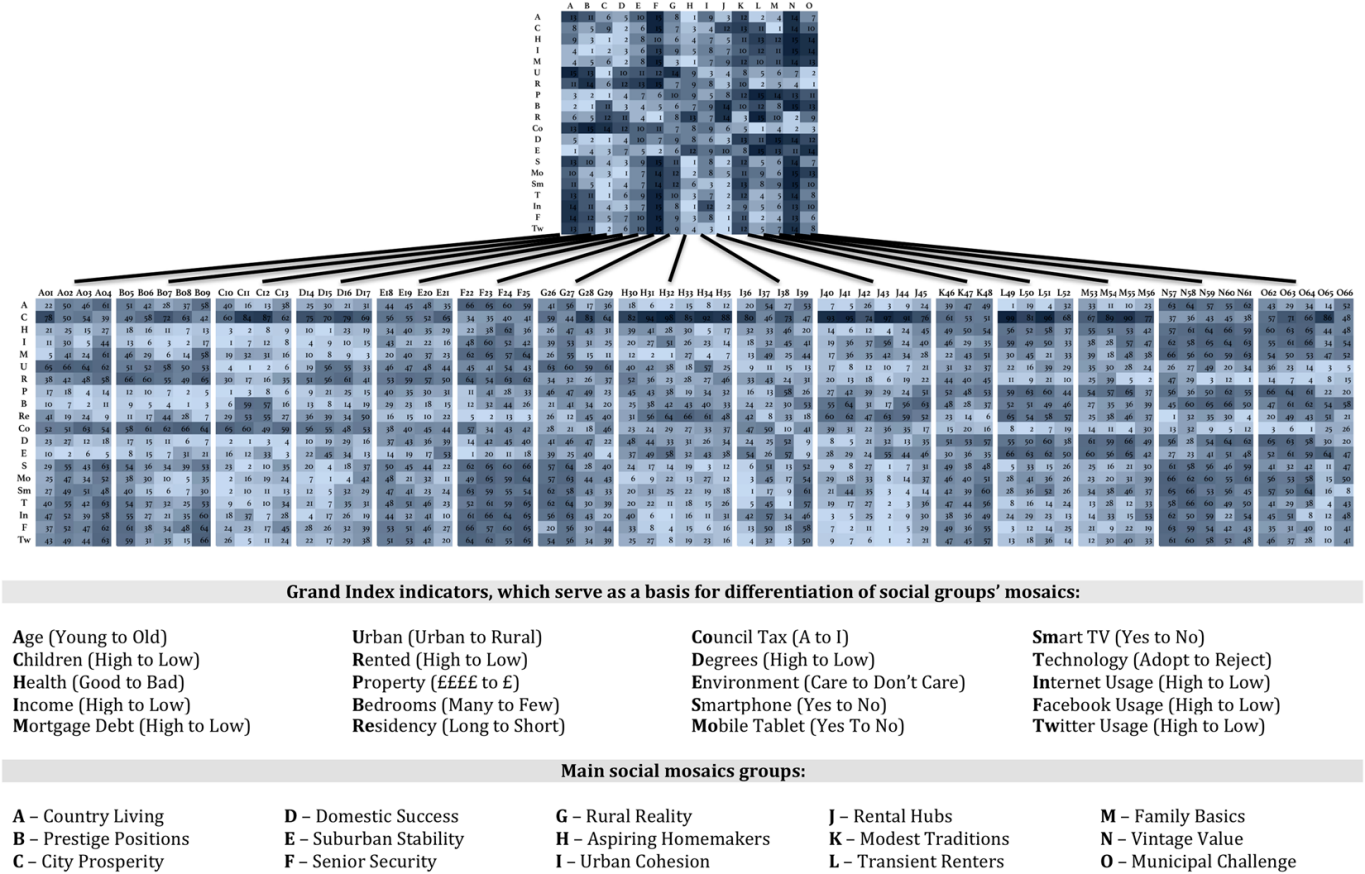
Structure of the Experian Mosaic Public Sector database.

**Figure 2:**
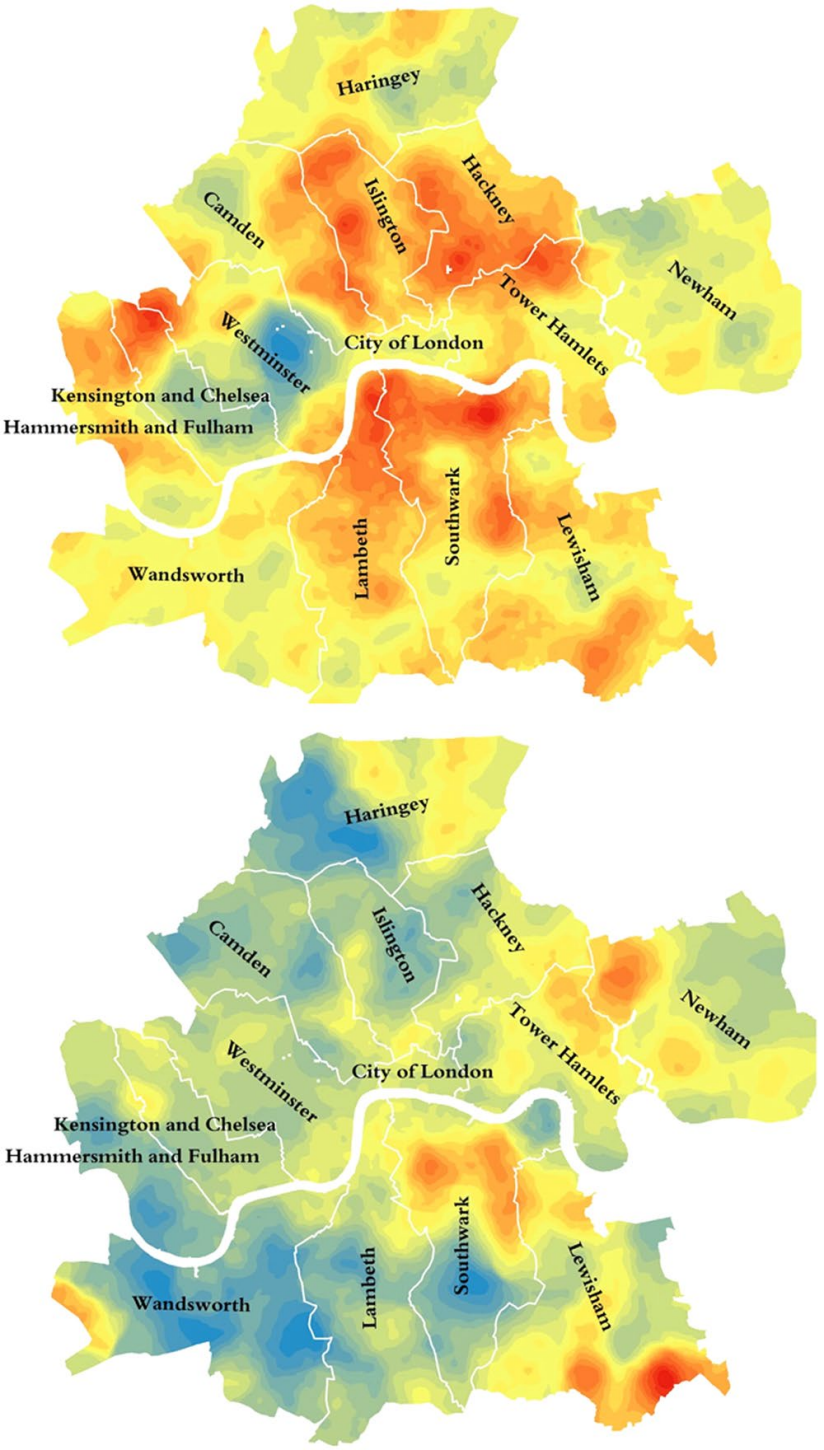
Medically- (top) and self- (bottom) diagnosed populations in Central London. Maps produced with the Experian Mosaic Public Sector data, using ArcGIS Desktop v.10: ESRI 2011. Redlands, CA: Environmental Systems Research Institute http://www.esri.com/software/arcgis/arcgis-for-desktop.

In search of the most integrative social bulk records to date, we decided to make use of a single geodemographic and lifestyle segmentation dataset, the Experian Mosaic Public Sector database (the detailed structure of the original components of the database is presented in the Supplementary Materials). This consists of UK postcode area aggregated records of traditional demographic indicators (such as gender and age) and various lifestyle choices (such as smoking, drinking, medical records or preferred communication channels) (Fig. 1). For this study, we selected records for the Central London geographical area, which is known to suffer the highest risk of diabetes as compared to the UK national average. An illustration of the distributions of both medically- and self-diagnosed populations across Central London is presented in (Fig. 2).

**Figure 3:**
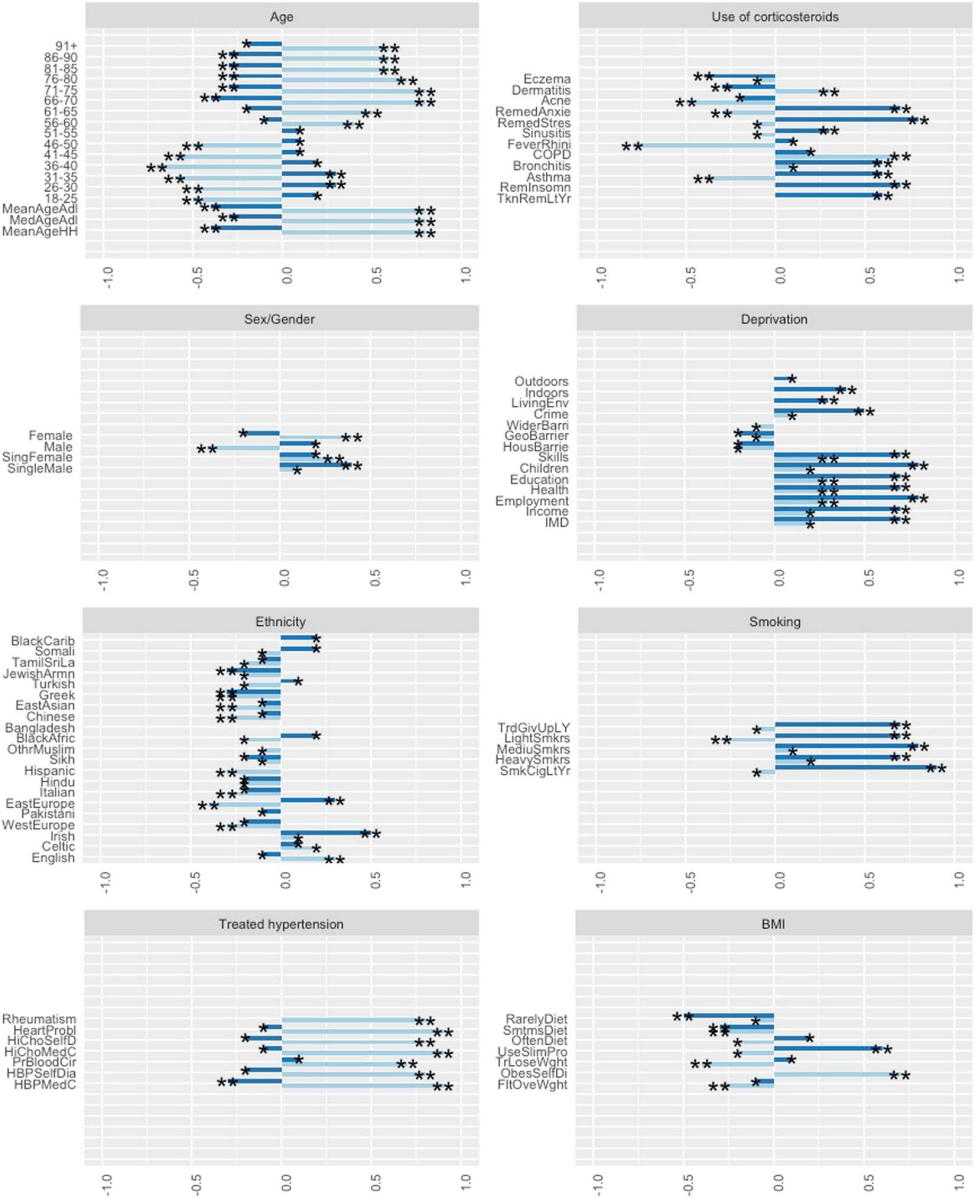
Medically- and self-diagnosed diabetes variables and EMPS risk variables, used by two main UK models Cambridge Risk Score and QDScore. Darker and lighter bars correspond to medically-diagnosed and self-diagnosed diabetes variables respectively. (*p < 0.1; **p < 0.05). This illustration was produced in R ggplot v2.1.0.

In order to extract the variables for modeling scenarios, we used a combined inventory of risk factors from two main UK models: Cambridge Diabetes Risk Score^12^ and QDScore^22^. Risk prediction models used by healthcare professionals are calculated from cohorts of non-diabetic individuals who are followed over time until some have developed diabetes and an ensuing assessment made based on risk factor measurements as to which were significant for those who developed the disease. The risk factors considered are: age, gender, ethnicity, Townsend score of deprivation, family history of diabetes, history of cardiovascular disease, smoking status, treated hypertension, use of corticosteroids and BMI. Variables, selected from Experian Mosaic datasets covered all the above risk categories, apart from family history of diabetes (genetic determinants of the disease). As the BMI risk factor was not available from the dataset, we used a selection of proxy variables instead: *‘Rarely Diet, ‘Sometimes Diet’ and ‘Often Diet’, ‘Use of Slimming Products’, ‘Trying to Lose Weight’*, *‘Self-diagnosed obesity’* and *‘Felt Overweight Last Year’*. The complete inventory of the candidate risk variables is presented in (Fig. 3), which also demonstrates strength and direction of their correlation with both medically- and self-diagnosed diabetes variables.

**Figure 4:**
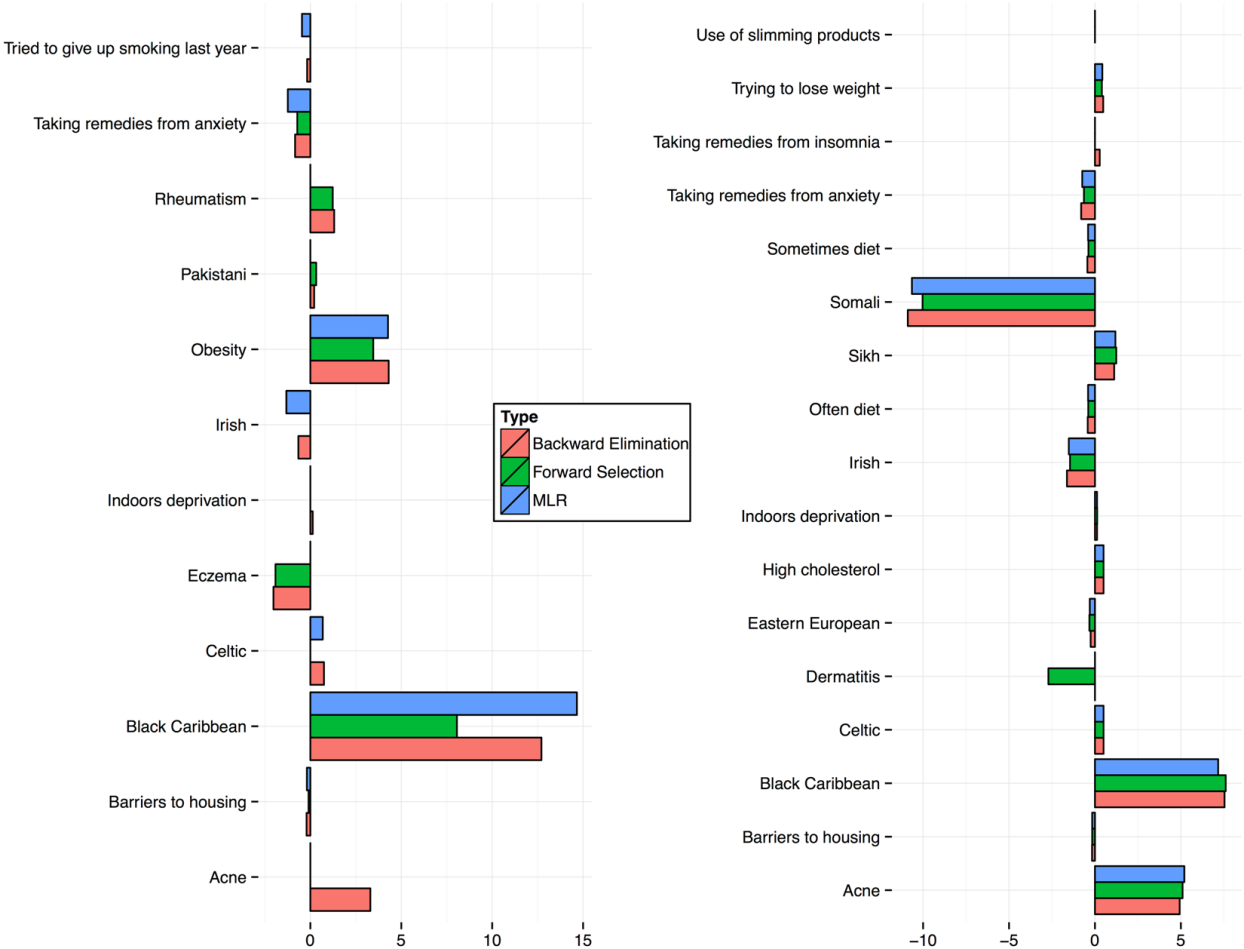
Candidate risk variables, constituting modeling scenarios ***without*** (left) and ***with*** (right) self-diagnosis variable. All regression coefficients presented have CI 95% and higher.

We applied both MLR and SMR regression methods for model scenarios, one of which comprised a ‘Self-diagnosed diabetes’ variable. Results of these simulations are presented in (Fig. 4) and in the Supplementary Materials document. Both scenarios demonstrated their consistency across all three models tested (standard MLR, Backward-AIC and Forward-AIC) in favour of the stronger forecast potential of the scenario derived from the inclusion of the self-diagnosis variable. Although statistically not significant as a direct regressor, it nevertheless can lead to a model structure where predictors reflect public interest (and potentially other tacit concerns) in the topic temporally much better than the scenario with traditional risk variables alone.

Here we also observe that the thematic composition of both scenarios is rather symmetrical (with the exception of the Smoking category, which is absent from the self-diagnosis (or information-seeking behaviour (ISB))) model scenario. Topic allocation of our results to such categories as Ethnicity, Poverty, Existing medical condition and Lifestyle, the primary role of which is currently being discussed in the research literature, illustrates that ISB model scenario indeed contains a bigger fraction of candidate risk factors, describing Lifestyle (27 per cent as compared to 8 per cent in the case of another model). This model has also somehow reduced the fraction of variables, associated with pre-existing Medical conditions (29 per cent as compared to 8 per cent in the case of another model), whilst categories of Ethnicity and Poverty remain fairly similar.

**Figure 5:**
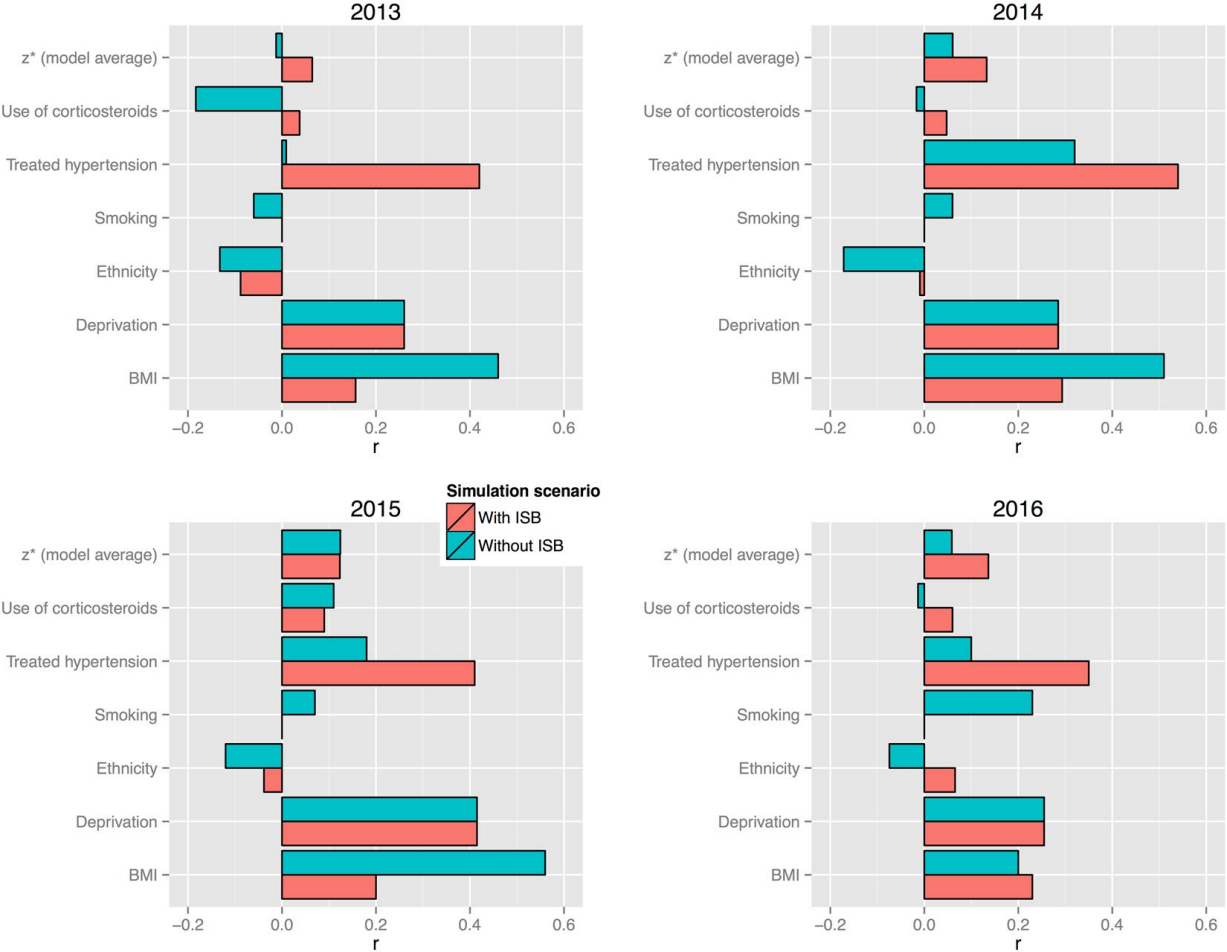
Correlation propensity between keywords, associated with the risk factors, contributing to model scenarios ***without*** and ***with*** self-diagnosis variable (ISB), and generic search term ‘diabetes’. Fisher-transformed *r*s indicate stronger correlation trends for the case of the modeling scenario, comprising self-diagnosis variable (‘With ISB’), leading to the conclusion that self-diagnosis can be seen as a mediator for highlighting more dynamic behavioural and lifestyle risk factors. Data source: Google Trends.

**Figure 6:**
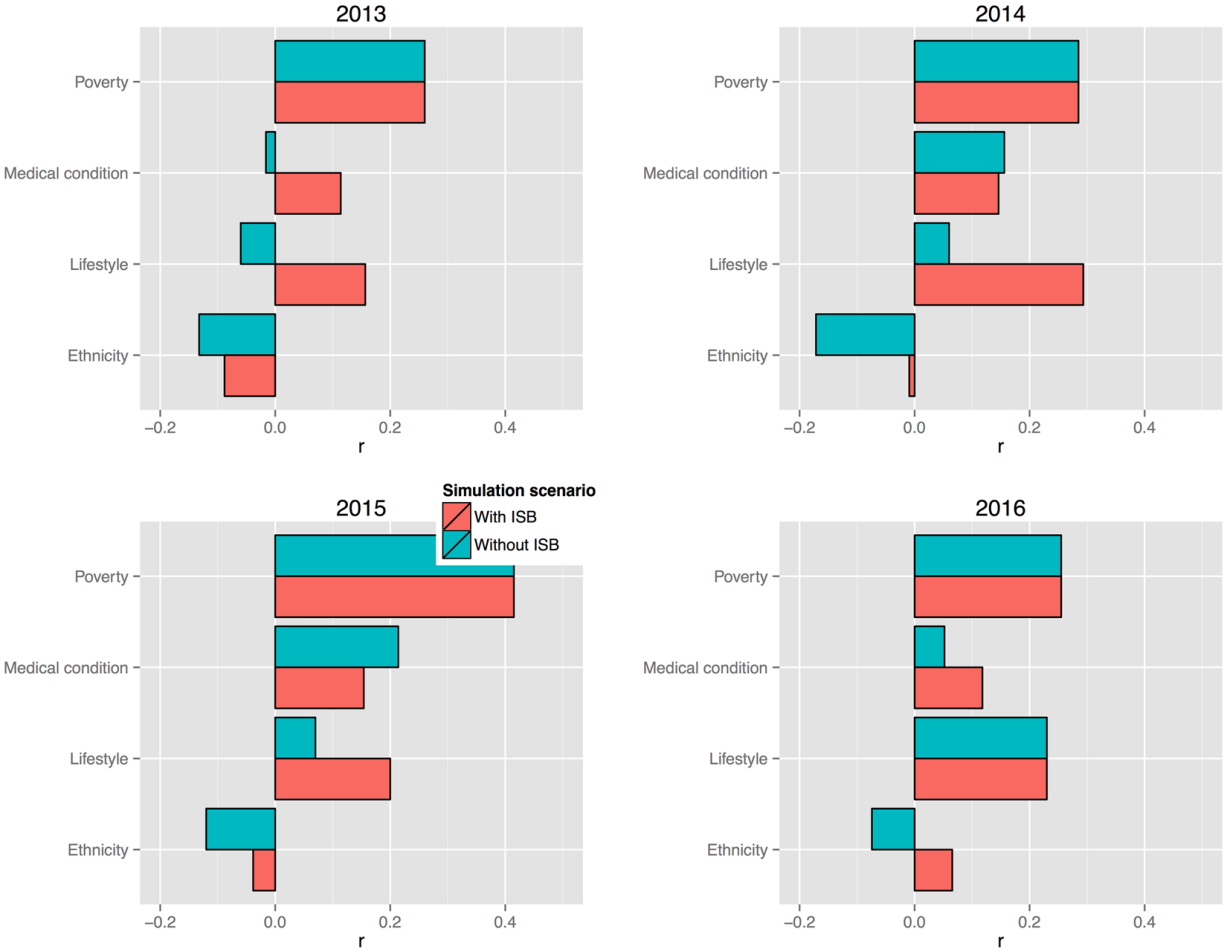
Correlation propensity between keywords, associated with the main risk topics and generic search term ‘diabetes’. Fisher-transformed *r*s indicate stronger correlation trends for the case of the modeling scenario, comprising self-diagnosis variable (‘With ISB’). Topics consist of the following risk factor components: *Poverty* (Without ISB): ‘Barriers to housing’, ‘Poor indoors living conditions’; *Medical condition* (Without ISB): ‘Obesity’, ‘Rheumatism’, ‘Acne’, ‘Eczema’, ‘Anxiety’; *Lifestyle* (Without ISB): ‘Smoking’; *Ethnicity* (Without ISB): ‘Pakistani’, ‘Irish’, ‘Celtic’, ‘Black Caribbean’; *Poverty* (With ISB): ‘Barriers to housing’, ‘Poor indoors living conditions’; *Medical condition* (With ISB): ‘Insomnia’, ‘Dermatitis’, ‘Acne’, ‘High cholesterol’, ‘Anxiety’; *Lifestyle* (With ISB): ‘Sometimes/Rarely diet’, ‘Use slimming products’, ‘Trying to lose weight’; *Ethnicity* (With ISB): ‘Irish’, ‘Somali’, ‘Sikh’, ‘Eastern European’, ‘Black Caribbean’. Data source: Google Trends.

We used Google Trends data and compared weekly fluctuation rates of the search keywords and word-combinations (a complete list is available in the Supplementary Materials) corresponding to the risk variable (e.g., *how to lose weight*) and the disease itself (*diabetes*). Figures 5 and 6 illustrate Fisher-transformed correlation coefficients (*r*s), aggregated to reflect the broad risk categories, currently utilized by medical practitioners and the mainstream media, respectively. Both figures demonstrate slightly stronger correlation advantage for the ISB model scenario, specifically for the risk factors, associated with lifestyle preferences and dietary habits, for example the Treated hypertension category in the ISB model is represented by the variable ‘High cholesterol’. Both models have the same factors, associated with the deprived living conditions: ‘Barriers to housing’ and ‘Poor indoors living conditions’.

Our simulation results demonstrate that application of Google Trends to chronic disease tracking can also be considered advantageous from the perspective of disease dynamics; It is becoming possible to monitor temporal growth or decrease of different risk factors in different geographic areas, which can provide valuable insights into the actual disease determinants (Type 2 diabetes in our case) and instigate further research into how they can be addressed.

## Discussion

Accurate behavioral surveillance of chronic diseases is a well-known challenge in healthcare^35^. Emerging data streams, also known as novel data streams (NDS) and alternative data (AD), comprising such signals as web search histories, social media updates or another user-generated content (UGC) are considered to hold promise for enhancing the capabilities of public health surveillance^28, 29, 35, 36^.

There are several ways these datasets could add value to existing disease surveillance: by increasing the timeliness of surveillance data, by improving resolution and data dissemination, by measuring unanticipated outcomes or by measuring aspects of transmission currently not captured by traditional surveillance data and methods (i.e., behaviour, perception). Normally, such datasets would not include official data sources, such as electronic health records, disease registries, emergency department visits or prescription history – although ready access to aggregated data from such sources is itself still novel in many health settings^28^.

From the perspective of epidemic studies, risk factors currently employed in the modeling of various lifestyle-related conditions fall into categories of traditional demographic variables. Such variables are routinely collected via surveys and other similar methods, which include user participation and observation. Survey methods have distinct advantages^37^ and, indeed, in this study, the self-diagnosis variable we introduced into the model is also collected in this way. However, it is being increasingly acknowledged that the proliferation and widespread adoption of online services such as search, purchasing of goods and social media opens up opportunities to improve on survey-based methods for capturing people’s behaviour and attitudes^38^. Often referred to as *post-demographics* in the field of digital anthropology^39^, the widespread adoption of these new population metrics still remain largely unaccounted for by epidemiological researchers. Nevertheless, the potential of using such naturally occurring data streams has been successfully demonstrated in a number of other fields, including natural hazards analytics^40^ and psychology^41^.

The advantages of the *hybrid modeling approach* we used here have already been argued by several authors^33, 34^. The contribution of self-diagnosis variables to the quality of disease surveillance demonstrated in this study could be explained by the fact that it can reflect such complex phenomena as disease perception^42^ and bodily experience of the emerging disease^43^. The trend for simplification (i.e., a commonly acknowledged *reductionist pathology* in general medicine during the last 200 years^44^) has led to the criticism that some emerging disease trends may be overlooked because they cannot be explained by the risk factors currently employed, or alternatively, lead to erroneous diagnoses if individuals have all the risk factors present, but no sign of an emerging illness. The importance of fine-tuning variables in such models therefore cannot be underestimated. Another question is how to appropriately select mediator variables for different illnesses. Several authors^45, 46^ hypothesize about the universal role of psychological conditions across the wide range of emerging bodily dysfunctions. In this study, we used the most obvious behavioural variable, which may reflect those psychological states, to lay out a method using additional variables that are widely mentioned in the medical research literature, but have not yet been empirically tested alongside other risk factors^47–50^.

## Methodology

### Data

Various commercial organizations (e.g., Experian, Acxiom, Qualtrics and Teradata) combine marketing records with other databases, thus providing value-added data services for research programmes conducting specific population studies. In this study, we make use of one such data service, provided by Experian, the Mosaic Public Sector v.1.04. Figure 1 in the Supplementary Material document illustrates the main topic variables constituting this data service. The Grand Index consists of 12 broadly defined categories (e.g., ‘Community safety’, ‘Health’, etc.), consisting of 1200 variables, which originate from a large number of databases. This dataset consists of 66 distinct social groups A1-O66, which are broadly classified according to age, socio-economic status, living arrangements and lifestyle. Figure 1 illustrates the detailed structure of the populations within the Experian Mosaic Public Sector dataset, each associated with distinct social, economic and behavioural profiles. The complete list of selected Mosaic variables per each Type 2 diabetes risk category, and how they correlate with both medically diagnosed and self-diagnosed diabetes variables, is presented in (Fig. 3).

We also collected weekly aggregated search terms from the Google Trends for the time period 2013–2016. The complete inventory of the risk- and disease-related keywords is provided in the Supplementary Materials section.

### Methods

Taking into consideration the linear relation between risk factors and diabetes in the currently used models, we tested several regression models (R stats v3.4.0) on both sets of independent variables (without and with the diabetes self-assessment variable) in order to extract the best statistically valid combinations. Prior to regression modeling, we used variance inflation factors (VIF) to identify collinearity among explanatory variables (Figs 2 and 3 in Supplementary Material section illustrate the Pearson correlation matrix and significance of correlation at p < 0.05, respectively). *VIF* calculations are fairly straightforward for interpretation: the higher the value, the higher the collinearity. For the case of a single explanatory variable, *VIF* is calculated using the R-squared value of the regression of that variable against the rest of the selected explanatory variables:

1 $$VI{F}_{n}=\frac{1}{1-{R}_{n}^{2}},$$

where *VIF* for a variable *n* is the reciprocal of the inverse *R* ^2^ from the regression.

*SMR* is a semi-automated routine for building a model by successively adding or removing variables based solely on the t-statistics of their estimated coefficients and is known to be useful in cases of testing large numbers of potential independent variables. The fact that variables we used are variants of the risk factors commonly accepted by healthcare professionals also substantially reduces the risk of their convergence on a poor model.

2 $$y={\beta }_{0}+{\beta }_{1}{x}_{i1}+{\beta }_{2}{x}_{i2}+{\beta }_{n}{x}_{in}+{u}_{i},$$

where *u*_*i*_ are values of an unobserved error term *u*. We follow the general assumptions that terms *u*_*i*_ are mutually independent and identically distributed, with $\bar{x}=0$ and constant variances.

The parameters ${\beta }_{0},{\beta }_{1}\ldots {\beta }_{n}$ are estimated using the least squares procedure, which minimizes the sum of squares of errors:

3 $$RSS=\sum _{i=1}^{n}{({y}_{i}-(\alpha +\beta {x}_{i}))}^{2}$$

The stepwise options allow starting the model-building process with no variables and proceeding forward (adding one variable at a time), or starting with all potential variables in the model and proceeding backward (removing one variable at a time). To run the models we used packages in the open source R software library that use a sequence of Akaike Information Criterion (AIC) values to add or eliminate a variable. During each iteration, the program performs the following calculations: for each variable currently in the model, it computes the t-statistic for its estimated coefficient, squares it and reports this as its *AIC-to-remove* statistic; for each variable not in the model, it computes the t-statistic that its coefficient would have if it were the next variable added, squares it and reports this as its *AIC-to-enter* statistic. At the next step, the program automatically enters the variable with the highest *AIC-to-enter* statistic, or removes the variable with the lowest *AIC-to-remove* statistic (for the detailed program outputs see Supplementary Material). The optimal model scenario uses the lowest *AIC* possible.

4 $$AIC=2k-2ln(L),$$

where *k* denotes estimated parameters in the model and *L* is the maximum value of the likelihood function for the model.

*F-value* was used to illustrate whether the model as a whole has a statistically significant predictive capability. Under the null hypothesis that the model has no predictive capability (all population regression coefficients simultaneously approach zero values), the F statistic follows an F distribution with *k* numerator degrees of freedom and *n* − *k* − 1 denominator degrees of freedom. The null hypothesis is rejected if the F ratio is large:

5 $$F=\frac{EV(MSR)}{UV(MSE)}$$

6 $$EV\,(ExplainedVariance)=\frac{{\sum }_{i}{n}_{i}{({\bar{x}}_{i}-\bar{x})}^{2}}{k-1},$$

where ${\bar{x}}_{i}$ denotes the sample mean in the ${i}^{th}$ group, ${n}_{i}$ is the number of observations in the *i* ^*th*^ group, $\bar{x}$ is the overall mean of the data and *k* is the total number of groups.

7 $$UV(UnexplainedVariance)=\frac{{\sum }_{ij}{({x}_{ij}-{\bar{x}}_{i})}^{2}}{n-k},$$

where ${\bar{x}}_{ij}$ is the *j* ^*th*^ observation in the *i* ^*th*^ out of *k* groups, and *n* is the overall sample size.

In order to compare temporal behaviour of the risk-related and disease-related search terms, extracted from Google Trends, we used standard Keyword Slope Formula:

8 $$KSF=\frac{rise}{run}=\frac{{y}_{2}-{y}_{1}}{{x}_{2}-{x}_{1}},$$

where *y* variables denote average search interest, measured during two distinct time points and *x* variables indicate number of time steps.

And, finally, in order to generate risk category specific notions of the correlations between risk- and disease related search terms, we apply Fisher *z* transformations to convert *r*s to *z*s, and then subsequently convert averaged *z* values back into *r*s^51^:

9 $$z=arctanh(r\mathrm{).}$$

## Electronic supplementary material

Supplementary Materials
