# Supplementary material for: Google Trends can improve surveillance of Type 2 diabetes
Source: Sci Rep. 2017 Jul 10;7:4993. doi: 10.1038/s41598-017-05091-9 (PMC5504026; doi:10.1038/s41598-017-05091-9)
Supplement: Supplementary file 1 — Supplementary Materials [file 41598_2017_5091_MOESM1_ESM.pdf]

Google Trends can improve surveillance of Type 2 diabetes

Nataliya Tkachenko[1,*], Sarunkorn Chotvijit[1], Neha Gupta[1], Emma Bradley[3], Charlotte Gilks[3], Weisi Guo[1], Henry Crosby[1], Eliot Shore[1], Malkiat Thiarai[1], Rob Procter[1,2], and Stephen Jarvis[1,2]

[1]Warwick Institute for the Science of Cities, University of Warwick, Coventry, CV4 7AL, UK
[2]Department of Computer Science, University of Warwick, Coventry, CV4 7AL, UK
[3]Experian, The Sir John Peace Building, Experian Way, NG2 Business Park, Nottingham, NG80 1ZZ, UK
*Corresponding Author: Nataliya.Tkachenko@warwick.ac.uk

SUPPLEMENTARY MATERIAL:

1. Database components constituting Experian Mosaic Public Sector variables (Fig 1)
2. Pearson correlation matrix between diabetes risk variables, selected from EMPS (Fig 2 and Fig 3)
3. Resulting metrics from Multilinear Regression (MLR) and Stepwise Multilinear Regression (SMR) (Table 1)
4. List of risk- and disease related keywords, used to extract weekly search volumes from the Google Trends

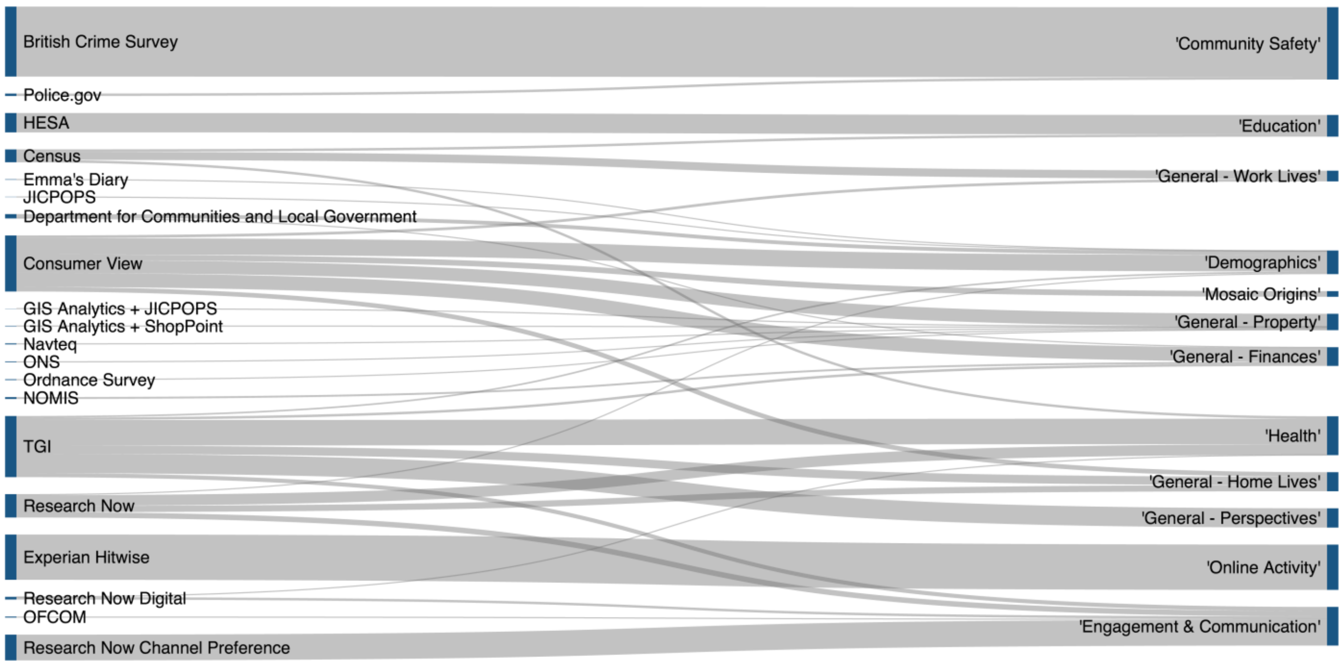

Fig 1. Database components constituting Experian Mosaic Public Sector variables

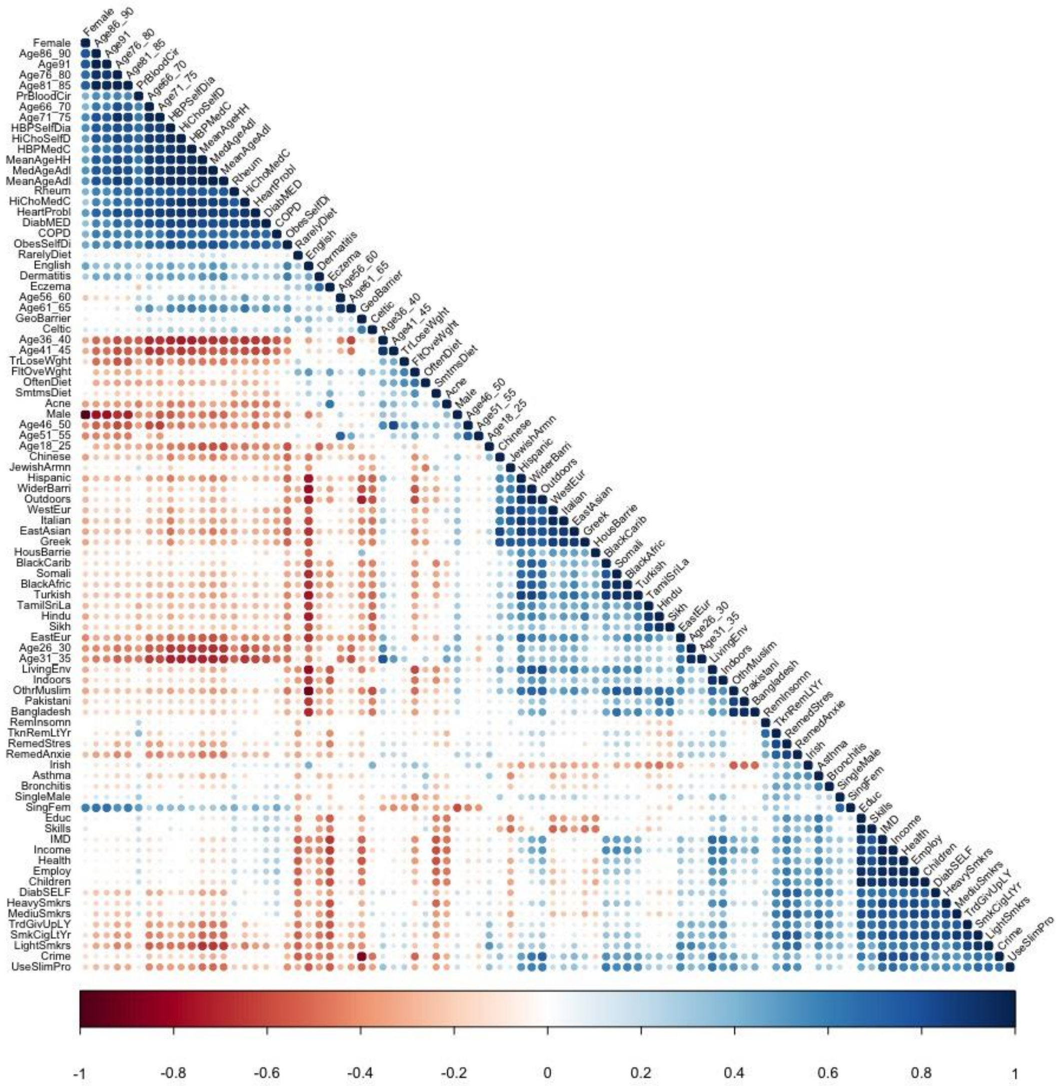

Fig 2. Pearson correlation matrix between diabetes risk variables, selected from EMPS. This illustration was produced in R corrgram v1.9.0

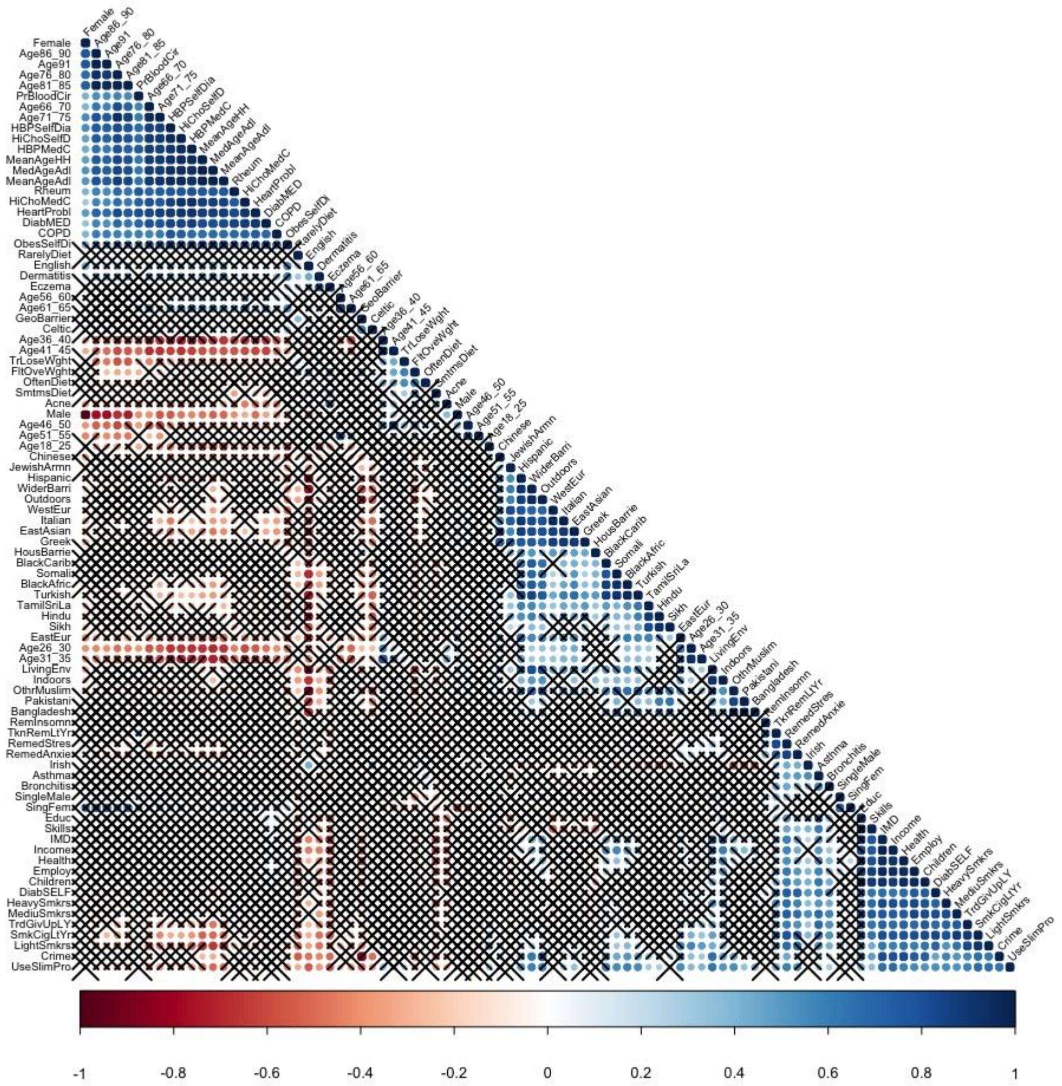

Fig 3. Pearson correlation matrix between diabetes risk variables, selected from EMPS: correlation significance at p<.05. This illustration was produced in R corrgram v1.9.0

	Without ISB			With ISB		
	F*	RSS	AIC**	F*	RSS	AIC**
Multilinear	9.83	4.05		26.25	1.51	
Backward AIC-Stepwise	31.65	2.56	53.15	45.52	1.09	16.88
Forward AIC-Stepwise	29.16	2.96	71.17	41.18	1.15	26.62

Table 1. Resulting metrics from regression scenarios demonstrate that the models with self-assessed diabetes variable (With ISB) perform better than traditional diabetes model variables (Without ISB). In this case, the Backward AIC-Stepwise model outperformed both Forward AIC-Stepwise and classic multilinear models, with the metrics being consistent across both scenarios.

(*p<.0001; **Akaike Information Criterion)

	Without ISB		With ISB	
	Group	Keywords	Group	Keywords
Disease	*'diabetes', 'Type 2 diabetes', 'diabetes mellitus'*			
Risk factors	Use of Corticosteroids	*'anxiety', 'eczema', 'acne'*	Use of Corticosteroids	*'anxiety', 'acne', 'insomnia'*
	Smoking	*'smoking', 'how to give up smoking', 'give up smoking'*	NA	
	Deprivation	*'deprivation', 'housing', 'cheap houses'*	Deprivation	*'deprivation', 'housing', 'cheap houses'*
	Ethnicity	*'pakistani', 'irish', 'celtic', 'black caribbean'*	Ethnicity	*'irish', 'somali', 'sikh', 'celtic', 'black caribbean', 'eastern european'*
	Treated Hypertension	*'rheumatism'*	Treated Hypertension	*'high cholesterol'*
	BMI	*'obesity'*	BMI	*'slimming products', 'how to lose weight', 'diet'*

Table 2. List of risk- and disease related keywords, used to extract weekly search volumes from the Google Trends. Data was extracted on the 7th of April 2017, for each risk group only the data from the search query with the highest propensity have been used in the analysis.
